# SUICIDES IN NEUROCOGNITIVE DISORDERS AND TRAUMATIC BRAIN INJURIES

**DOI:** 10.1192/j.eurpsy.2023.251

**Published:** 2023-07-19

**Authors:** T. Talaslahti, M. Ginters, H. Kautiainen, R. Vataja, A. Palm, H. Elonheimo, J. Suvisaari, H. Koponen, N. Lindberg

**Affiliations:** 1 Psychiatry, Helsinki University Hospital; 2 Psychiatry, University of Helsinki, Helsinki; 3 Kuopio University Hospital, Kuopio; 4 Folkhälsan Research Center; 5 Finnish Institute for Health and Welfare, Helsinki, Finland

## Abstract

**Introduction:**

Depression, anxiety and lack of impulse control are common neuropsychiatric symptoms in neurocognitive disorders and have been strongly associated with suicidality.

**Objectives:**

The aim of this study was to explore suicide rates in three major neuropsychiatric conditions including various degenerative neurocognitive disorders (DND), alcohol related neurocognitive disorders (ARND), and traumatic brain injuries (TBI).

**Methods:**

The register cohort data of 231 817 patients with a diagnosis of degenerative dementias, ARND, or TBI, and their mortality data were collected from Finnish nationwide registers between 1998 and 2018. We calculated incidences of suicides, types of suicides, and suicide rates compared with the age- and sex matched general population (Standardized Mortality Ratio, SMR).

**Results:**

In fifteen years since diagnosis, 0.3% (95% CI: 0.2 to 0.5) of patients with DND, 1.1% (0.7 to 1.8) of patients with ARND, and 1.0% (0.7 to 1.3) of patients with TBI died from suicide (**Figure**). Men died from suicide more often than women [58.9 (51.3 to 67.4) vs. 9.8 (7.5 to 12.5) per 100 000 person-years]. Of all three groups of patients, the highest number of suicides per 100 000 was in ARND (98.8; 65.1 to 143.8), then in TBI (82.0; 62.4 to 105.8), and then in DND (21.2; 18.3 to 24.5). The most common cause of death per 100 000 person-years was self-inflicted injury by hanging, strangulation or suffocation and drowning (12.4, 10.3 to 14.8), the second highest incidence self-inflicted poisoning (5.7, 4.3 to 7.4), and then self-inflicted injury by firearms, explosives, smoke, fire, flames, steam, hot vapours or hot objects (4.7, 3.4 to 6.2). The SMRs (95% CI) in the DND group were 1.31 (1.13 to 1.51) for the whole group, 1.21 (0.90-1.62) for women, and 1.34 (1.14-1.58) for men. The SMRs in the ARND group were 3.69 (2.53-5.38), 5.05 (1.90 to 13.46), and 3.52 (2.34 to 5.30), and in the TBI group 2.99 (2.31 to 3.86), 5.68 (3.22 to 10.00), and 2.66 (2.00 to 3.55), respectively.

**Image:**

**Figure:**
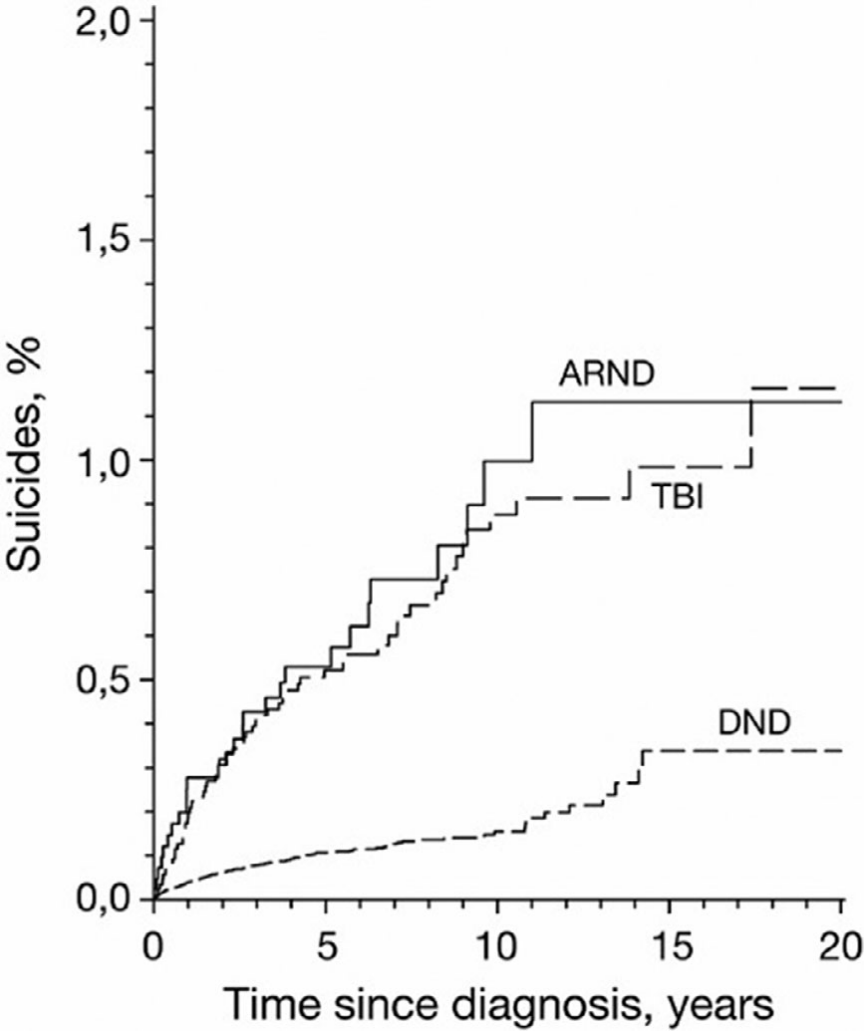

**Conclusions:**

Suicide rates were higher in all three patient groups compared with the same-aged general population. Risk for death from suicide remained elevated for more than ten years after the initial diagnosis. Men committed more suicides than women, but there was no difference between sexes in comparison with the age-matched general population. The suicide methods were mostly violent.

**Disclosure of Interest:**

T. Talaslahti Grant / Research support from: Helsinki University Hospital, grant no 212 9003, M. Ginters: None Declared, H. Kautiainen: None Declared, R. Vataja: None Declared, A. Palm: None Declared, H. Elonheimo: None Declared, J. Suvisaari: None Declared, H. Koponen: None Declared, N. Lindberg: None Declared

